# Soil heavy metals in a typical coal mining cluster of Northern China: Pollution, source apportionment, and eco-health risks

**DOI:** 10.1016/j.isci.2026.116835

**Published:** 2026-07-16

**Authors:** Dandan Du, Lijing Fang, Shuyu Yu, Yanying Bai, Yusheng Hao, Hao Li

**Affiliations:** 1 College of Water Conservancy and Civil Engineering, Inner Mongolia Agricultural University, Hohhot, Inner Mongolia Autonomous Region 010018, China; 2 State Key Laboratory of Water Engineering Ecology and Environment in Arid Area, Hohhot 010018, China; 3 Research Center for High-Quality Economic Development of the Yellow River Basin in Inner Mongolia, Hohhot, Inner Mongolia Autonomous Region 010070, China; 4 Resource Utilization and Environmental Protection Coordinated Development Academician Expert Workstation in the North of China, Inner Mongolia University of Finance and Economics, Hohhot, Inner Mongolia Autonomous Region 010070, China; 5 Office of Scientific Research, Inner Mongolia University of Finance and Economics, Hohhot, Inner Mongolia Autonomous Region 010070, China

**Keywords:** coal mining area, soil heavy metals, PMF source apportionment, potential ecological risk, health risk assessment

## Abstract

Coal mining activities can alter soil heavy-metal accumulation and risk patterns. To identify pollution characteristics, sources, and ecological and health risks, 88 surface soil samples from Tongchuan Town, Ordos City, were analyzed using enrichment factor, potential ecological risk index, positive matrix factorization, soil physicochemical analysis, and Monte Carlo-based health risk assessment. Heavy-metal concentrations were generally low and regional pollution risk was controllable, although Cd exceeded the agricultural soil risk screening value at some sites. Cd and Hg showed local enrichment and dominated ecological risk, with over 60% of sites at moderate or higher risk. Industrial-traffic sources contributed the most (44.09%), followed by natural sources (34.06%), coal-related emission-atmospheric deposition (16.35%), and mining disturbance-agricultural inputs (5.50%). Soils were strongly alkaline, with low organic matter and nitrogen and potassium deficiencies. Health risks were acceptable; oral ingestion was dominant, Cr and As were key contributors, and children were more sensitive.

## Highlights

• Soil metal pollution was generally low, but local Cd accumulation occurred
• Cd and Hg drove ecological risk; industrial-traffic mixed source contributed most
• Soils were alkaline, with low organic matter and nutrient deficiency
• Health risks were acceptable, but children had higher risks than adults

## Graphical abstract

**Figure:**
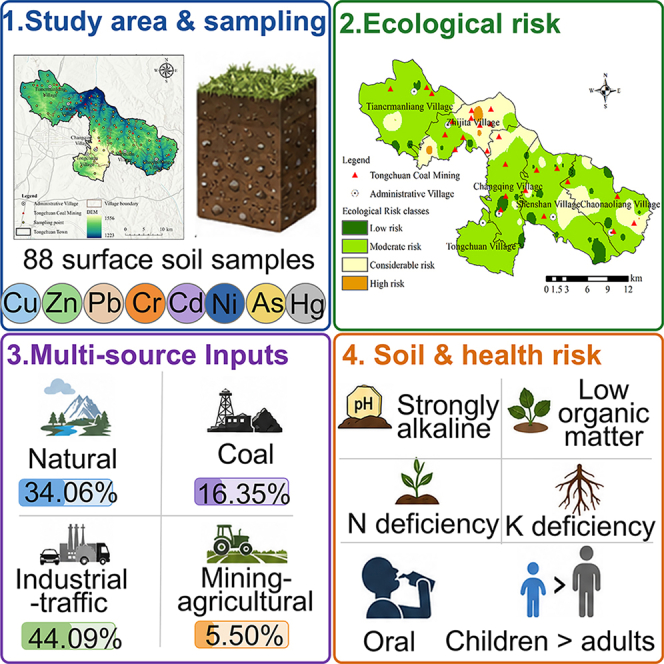

## Teaser

Soil science; Environmental science; Pollution; Soil ecotoxicology

## Introduction

Soil is an essential component of terrestrial ecosystems, and its environmental quality is directly related to regional ecological security, sustainable land use, and human health.^1^ In areas of intensive coal mining, activities such as coal extraction, washing, storage, and transportation can alter the input, migration, and accumulation of heavy metals in soils through pathways, including dust deposition, leaching from coal gangue, seepage of mine drainage, and surface disturbance, thereby leading to soil function degradation, accumulation of ecological risks, and increased human exposure risks.^2^ Previous studies have shown that elements, such as Cd, Hg, As, and Cr in soils from coal mining areas often exhibit varying degrees of enrichment, and highly toxic elements are frequently the major drivers of ecological and health risks.^3^^,^^4^^,^^5^ Therefore, clarifying the pollution characteristics, dominant sources, and risk effects of soil heavy metals in coal mining areas is an important basis for developing pollution control and ecological restoration strategies.

In recent years, research on soil heavy-metal pollution in coal mining areas has gradually developed a basic framework centered on pollution identification, source apportionment, and risk assessment.^6^^,^^7^^,^^8^^,^^9^^,^^10^^,^^11^^,^^12^^,^^13^ Existing studies generally employ methods, such as enrichment factors, potential ecological risk indices, health risk models, and receptor models to identify and quantify pollution levels, potential sources, and risk status. However, previous work has focused more on “how severe the pollution is” and “where the sources come from,” while paying insufficient attention to how different sources further shape the spatial patterns of ecological and health risks through spatial heterogeneity. This gap is particularly evident in intensively mined coalfield areas, where mining disturbance, industrial and traffic activities, residential aggregation, and other anthropogenic processes are superimposed. Under such multi-source input conditions, the spatial differentiation of soil heavy metals and the mechanisms underlying their risk responses remain insufficiently understood.

Ordos is one of the most important coal energy bases in China, and high-intensity resource exploitation has made soil heavy-metal pollution in mining areas and their surroundings increasingly prominent. Previous studies have shown that elements such as Cd and Hg are relatively heavily polluted in soils around different mining areas in Ordos and that both ecological and health risks exhibit clear anthropogenic driving characteristics.^14^^,^^15^^,^^16^ However, existing studies have mostly focused on individual mines, waste dumps, or local functional zones, and the research objects remain relatively fragmented, making it difficult to reveal the overall impacts of concentrated and large-scale coal mining on the regional soil environment. Compared with a single mining area or local functional zone, the township-scale system under intensive coal mining simultaneously bears multiple processes, including mining disturbance, industrial and traffic activities, and residential aggregation, and therefore represents an important research unit for identifying the relationships among multi-source inputs, spatial differentiation, and risk responses of soil heavy metals. Thus, extending the research object to a township-scale composite system is not simply a matter of enlarging the study scale but rather aims to elucidate the formation and risk evolution of heavy metal pollution from the perspective of a coupled human-environment system.

**Figure 1:**
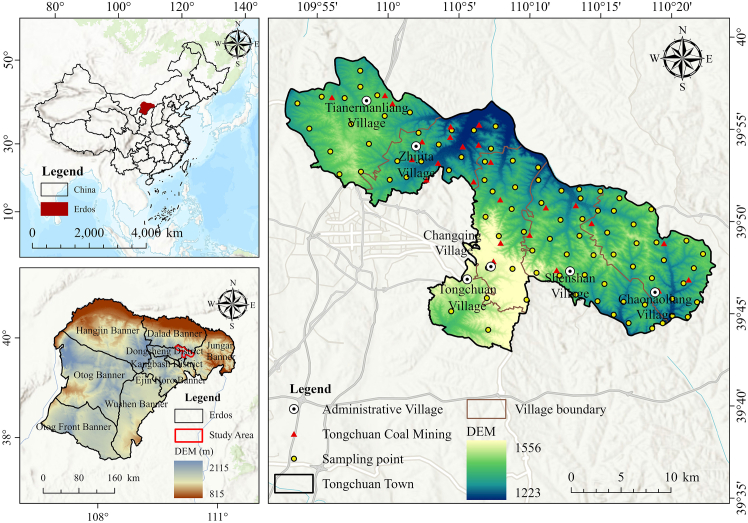
Distribution map of sampling points

Accordingly, this study takes Tongchuan Town in Ordos City (Figure 1), a typical intensive coal mining area, as the study area and focuses on the following questions: (1) What are the concentration distribution, enrichment levels, and spatial heterogeneity characteristics of soil heavy metals under the combined influence of intensive coal mining and township-scale human activities? (2) What are the dominant sources of different heavy metals and their spatial correspondences, particularly how can the relative roles of mining activities, industrial and traffic activities, and other anthropogenic inputs be distinguished within a multi-source composite system? (3) How do different pollution sources further drive the differentiated responses of ecological and health risks? To address these questions, based on surface soil sampling and laboratory analyses, this study systematically identifies the pollution characteristics, source structure, and risk-response relationships of soil heavy metals by integrating enrichment factor analysis, potential ecological risk assessment, soil fertility evaluation, positive matrix factorization (PMF) source apportionment, and probabilistic health risk assessment based on Monte Carlo simulation. Compared with previous studies, the main features of this study are as follows: first, it moves beyond source identification to a comprehensive analysis of the “source-spatial differentiation-risk response” framework; second, it uses a township-scale composite system under intensive coal mining as the research unit to characterize the overall pattern of soil heavy-metal pollution under multiple overlapping sources; third, it introduces Monte Carlo simulation into health risk assessment to probabilistically characterize the uncertainty of exposure parameters, thereby improving the robustness of risk evaluation. The results can provide a scientific basis for precise pollution control, risk-based zoning management, and ecological security protection in intensive coal mining areas.

## Results

### Characteristics of soil heavy-metal concentrations

The concentrations of heavy metals in the soils of Tongchuan Town were generally low, yet they predominantly exceeded or approached the soil background values of Inner Mongolia^17^ (Table 1). The mean concentrations followed the descending order of Cr > Zn > Ni > Cu > Pb > As > Cd > Hg, indicating varying degrees of accumulation for all eight heavy metals in the soil. Notably, the maximum concentrations of Cd, Hg, and Cr reached 17.8, 15, and 3.58 times their respective background values, demonstrating significant accumulation effects. The average concentrations of elements such as Zn and Pb were close to or slightly higher than the background levels. Compared with the risk screening values for soil contamination of agricultural land (2018), the mean concentrations of all elements were substantially lower. Only the maximum value of Cd (0.89 mg kg^−1^) exceeded the screening threshold, suggesting that while the overall regional pollution risk is low, localized Cd contamination risks persist. The coefficients of variation revealed that Cd (103.88%) and Hg (127.88%) exhibited the strongest spatial variability, indicating pronounced influence from anthropogenic activities such as mining and transportation. For the remaining elements, coefficient of variation (CV) values mostly ranged between 12% and 25%, representing moderate variability. Furthermore, the skewness and kurtosis of most elements were positive, suggesting enrichment at a limited number of sampling sites. Ni displayed the lowest CV (12.37%), reflecting a relatively uniform spatial distribution and minimal anthropogenic disturbance.^18^

**Table 1:** Statistical analysis of heavy metal content in soils of the study area

Heavy metal	Minimum (mg·kg^−1^)	Maximum (mg·kg^−1^)	Mean (mg·kg^−1^)	SD (mg·kg^−1^)	Skewness	Kurtosis	CV(%)	Background value of Inner Mongolia (mg·kg^−1^)	Risk screening value for agricultural land (mg·kg^−1^)
Cu	8.92	36.49	19.07	4.75	1.42	2.90	24.88	14.40	100
Zn	37.02	112.75	59.47	14.79	1.35	1.95	24.88	59.10	300
Pb	10.43	23.22	18.04	2.25	−0.29	0.98	12.47	17.20	170
Cr	71.16	148.15	115.84	14.89	−0.30	0.65	12.85	41.40	250
Cd	0.01	0.89	0.17	0.18	2.20	5.54	103.88	0.05	0.6
Ni	23.89	44.60	31.68	3.92	0.89	1.38	12.37	19.50	190
As	2.45	16.04	9.89	2.34	−0.37	0.86	23.68	7.50	25
Hg	0.01	0.60	0.11	0.13	1.82	2.46	127.88	0.04	3.4

### Evaluation of soil heavy-metal pollution

**Figure 2:**
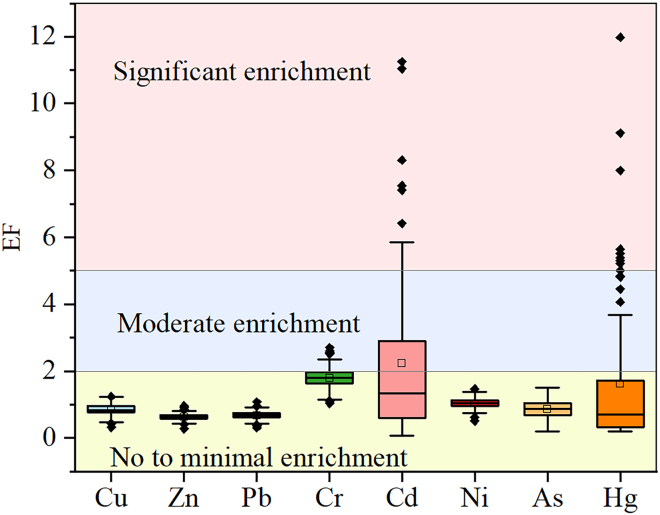
Evaluation results of enrichment factors for heavy metals in soil

The enrichment factors for all heavy metals were generally at a low enrichment level, with mean enrichment factor (EF) values following the descending order: Cd > Cr > Hg > Ni > As > Cu > Pb > Zn (Figure 2). The median EF values for Cu, Zn, Pb, Ni, and As were all near 1 with low degrees of dispersion, indicating that these elements were not significantly influenced by exogenous inputs. The EF of Cr was slightly higher than that of other elements, with a median near 1.5; however, it remained consistently below 2, representing only minor enrichment. The enrichment characteristics of Cd and Hg were the most prominent. According to the EF pollution classification standards, Cd and Hg showed relatively severe enrichment levels. For soil Cd, samples categorized under significant enrichment and moderate enrichment accounted for 13.64% and 25.00%, respectively. Most sampling sites reached the weak-to-moderate enrichment level, with localized areas exhibiting significant enrichment (EF > 5). For soil Hg, samples showing significant and moderate enrichment accounted for 10.23% and 13.64%, respectively. Although the overall median for Hg was not high, numerous discrete high-value points were observed, with some sites also falling into the significant enrichment range. In summary, the localized enrichment of Cd and Hg was the most evident in the study area, suggesting that their accumulation is significantly influenced by anthropogenic activities within the region. In contrast, the EF values for Ni, As, Cu, Pb, and Zn were generally low, remaining within the range of no enrichment to weak enrichment, which implies that their distribution is primarily controlled by natural factors.^19^^,^^20^

### Evaluation of potential ecological risk index

**Figure 3:**
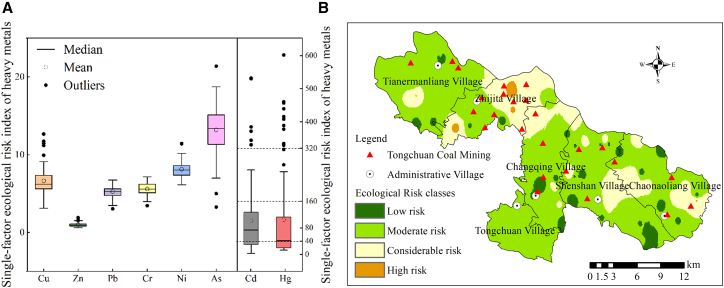
Spatial distribution of potential ecological risk (A) Potential ecological risk assessment for single heavy metals. (B) Integrated potential ecological risk (RI).

The potential ecological risk index for individual heavy metals (*Eᵢ*) exhibited significant differences (Figure 3A and Table 2). The mean values followed the descending order: Hg > Cd > As > Ni > Cu > Cr > Pb > Zn. Except for Cd and Hg, the *Eᵢ* values for Cu, Zn, Pb, Cr, Ni, and As were all below 40, representing a low ecological risk. In contrast, Cd (*Eᵢ*: 3.47–531.63) and Hg (*Eᵢ*: 12.50–601.50) showed higher risk levels. Specifically, 27.27%, 9.09%, and 6.82% of the samples for Cd, and 14.77%, 7.95%, and 12.50% for Hg, reached the levels of considerable, high, and very high risk, respectively. The average *Eᵢ* values for Cd and Hg were 102.01 and 105.17, respectively. Both elements displayed strong dispersion and a high concentration of outliers, indicating that Cd and Hg are the primary contributors to the regional potential ecological risk.

**Table 2:** Evaluation results of potential ecological risk index for soil heavy metals

Individual potential ecological risk index	Heavy metal	*E*_*j*_ Range	*E*_*j*_ mean	Percentage of different risk levels(%)				
				Low	Moderate	Considerable	High	Very high
*E*_*j*_	Cu	3.10–12.67	6.62	100	0	0	0	0
	Zn	0.63–1.91	1.01	100	0	0	0	0
	Pb	3.03–6.75	5.24	100	0	0	0	0
	Cr	3.44–7.16	5.60	100	0	0	0	0
	Cd	3.47–531.63	102.01	35.23	21.59	27.27	9.09	6.82
	Ni	6.12–11.44	8.12	100	0	0	0	0
	As	3.27–21.39	13.18	100	0	0	0	0
	Hg	12.50–601.50	105.17	47.73	17.05	14.77	7.95	12.50

Integrated potential ecological risk index (*RI*)	*RI* Range	*RI* Mean	Percentage of integrated risk levels (%)				
			Low	Moderate	Considerable	High	Very high
	61.99–714.6	246.96	34.10	39.77	20.45	5.68	–

The integrated potential ecological *RI* for the study area ranged from 61.99 to 714.6, with a mean value of 246.96, indicating an overall moderate-to-high risk level. Samples categorized as moderate risk or higher accounted for over 60% of the total, suggesting that ecological environmental pressure is prominent in localized areas (Table 2). In terms of spatial distribution (Figure 3B), the study area was generally dominated by low-to-moderate risk, with only a few localized patches of moderate to high risk, mainly distributed around mining sites and coal-washing facilities. Considering the spatial distribution of the mining areas shown in the figure, the high-risk zones exhibited a certain spatial correspondence with coal mining activities, suggesting that human activities may have played an important role in shaping the local ecological risk pattern. In summary, the potential ecological risk of soil heavy metals in the study area showed distinct heterogeneity across different elements and spatial locations. While the overall ecological risk remained manageable, Hg and Cd were identified as the key factors driving the localized potential ecological risks.

### Source apportionment using the PMF model

**Figure 4:**
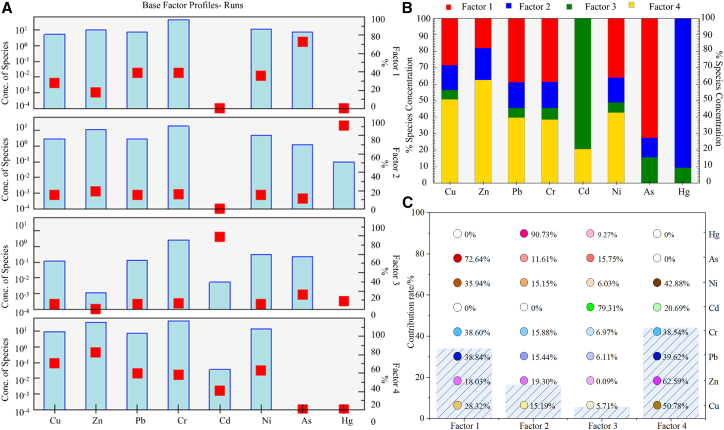
Source contribution rates of pollution sources (A) Contribution characteristic map of soil heavy metals based on PMF source apportionment. (B) Proportional contribution chart of soil heavy-metal source factors. (C) Source contribution rates of pollution sources for each soil element.

The PMF model resolved four pollution source factors, each showing distinct fingerprint characteristics and contribution patterns for different heavy metals (Figure 4). In factor 1, As exhibited the most prominent contribution, accompanied by certain proportions of Cr, Pb, and Ni, forming a multi-element assemblage dominated by As. Considering the geological background of the study area and the occurrence characteristics of these elements, this factor mainly reflects natural inputs controlled by parent material weathering and pedogenesis and can therefore be identified as a natural source. Factor 2 was almost entirely dominated by Hg, exhibiting a typical Hg-controlled signature, which suggests a relatively independent source origin. Combined with the coal-mining background of the study area and the spatial distribution of Hg, this factor is more likely associated with processes, such as coal mining, coal washing and processing, coal combustion emissions, and atmospheric deposition and can thus be defined as a coal-related emission-atmospheric deposition source. Factor 3 was overwhelmingly dominated by Cd, while contributions from other elements were relatively weak, indicating that this factor primarily represents a characteristic source of Cd. Further consideration of the spatial distribution of Cd showed that its high-value areas were mainly concentrated in and around mining sites and displayed good spatial correspondence with the distribution of coal mines, indicating that mining disturbance processes, including coal extraction, coal gangue accumulation, transport-related dust, and weathering-leaching processes, played an important role in Cd enrichment. Meanwhile, agricultural inputs such as fertilization and irrigation may also have contributed to local Cd accumulation. Therefore, factor 3 should not be simply attributed to a single agricultural source but is more appropriately defined as a mining disturbance-agricultural input mixed source. Factor 4 was mainly characterized by Cu, Zn, Pb, and Ni, together with a certain amount of Cr, showing a typical multi-metal composite enrichment pattern. This indicates that it mainly reflects combined anthropogenic influences, such as industrial emissions, traffic-related wear, and urban non-point source inputs, and can therefore be identified as an industrial-traffic mixed source (Figure 4A). The overall contribution rates of the four factors indicated that factor 4 was the dominant source, followed by factor 1, whereas factors 2 and 3 contributed less to total pollution overall but showed clear dominance for Hg and Cd, respectively (Figure 4B). The source contribution proportions for individual elements further confirmed these interpretations: the industrial-traffic mixed source contributed the most overall, mainly affecting Cu and Zn and also exerting relatively strong influences on Pb, Cr, and Ni; the natural source contributed most prominently to As and also showed certain contributions to Cr and Pb; the coal-related emission-atmospheric deposition source almost entirely controlled the source of Hg; and the mining disturbance-agricultural input mixed source primarily controlled the source of Cd (Figure 4C). Overall, the sources of soil heavy metals in the study area exhibited clear multi-source composite characteristics, with As mainly controlled by natural background, Hg mainly associated with coal-related activities, Cd characterized by a composite source dominated by mining disturbance with additional agricultural input, and Cu, Zn, as well as part of Pb, Cr, and Ni mainly influenced by the industrial-traffic mixed source.

**Figure 5:**
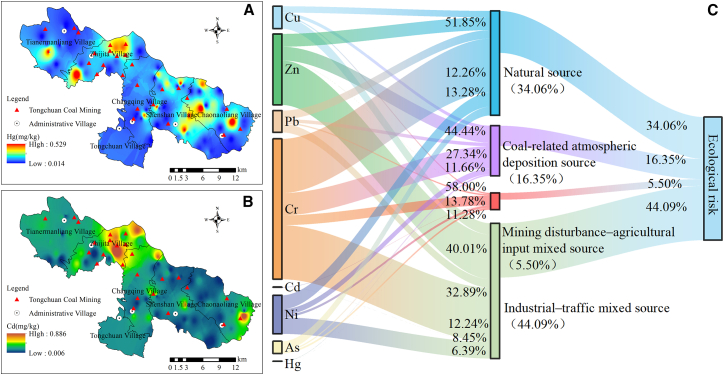
Spatial distributions of Hg and Cd and contributions of different pollution sources to the integrated ecological risk (A) Spatial distribution of Hg. (B) Spatial distribution of Cd. (C) Contributions of different pollution sources to the integrated ecological risk.

Based on the source apportionment results shown in Figure 4, and further combined with ecological risk contributions and spatial distribution patterns, it is evident that different sources play markedly different roles in driving the integrated ecological risk (Figure 5). Among them, the industrial-traffic mixed source contributed the most to the integrated ecological risk (44.09%), followed by the natural source (34.06%), while the coal-related emission-atmospheric deposition source and the mining disturbance-agricultural input mixed source contributed 16.35% and 5.50%, respectively (Figure 5C), indicating that the ecological risk of soil heavy metals in the study area was overall dominated by anthropogenic sources. In terms of spatial pattern, high Hg concentrations were mainly distributed in and around the mining areas and showed a certain diffusive enrichment in some local residential activity zones (Figure 5A), suggesting that coal-related activities played a significant role in Hg accumulation. High Cd concentrations were mainly concentrated in the mining areas and their surroundings (Figure 5B), further indicating that, in addition to local agricultural inputs, its enrichment was also closely related to mining disturbance processes, such as coal extraction, coal gangue accumulation, transport-related dust, and weathering-leaching. From the perspective of element-specific risk contributions, Cr was mainly controlled by the natural source (51.85%), whereas Zn and Ni were primarily influenced by the industrial-traffic mixed source, with contributions of 44.44% and 40.01%, respectively (Figure 5C). Overall, Figure 5 demonstrates from both spatial distribution and risk contribution perspectives that the industrial-traffic mixed source and coal-related activity sources were the major drivers of heavy-metal ecological risk in the study area. Future pollution control and ecological restoration efforts should therefore focus on mining areas and their surrounding disturbed zones, as well as areas strongly affected by industrial and traffic activities, with classified prevention and zone-specific management targeting key elements, such as Hg, Cd, As, and Cr.

### Soil physicochemical properties and their potential influence on heavy-metal behavior

**Figure 6:**
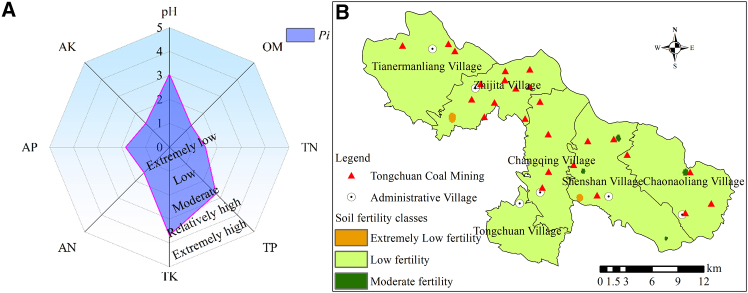
Spatial distribution of soil fertility quality (A) Soil nutrient quality radar chart. (B) Spatial distribution of soil fertility.

The topsoil in the study area was generally strongly alkaline (pH 7.44–8.86, with a mean of 8.47; 54.55% of the samples had pH > 8.5), which may be related to salt accumulation. Overall, soil nutrients were relatively low, as reflected by low organic matter and nitrogen contents (organic matter: 3.19–20.14 g/kg; mean total nitrogen: 0.61 g kg^−1^, with 76.14% of the samples classified as deficient or extremely deficient), and available nitrogen was also at a low level. Total phosphorus was generally moderate to relatively abundant (89.77% of the samples reached grade III or above), whereas 44.32% of the samples were still deficient or extremely deficient in available phosphorus (AP). Total potassium was relatively sufficient, while available potassium was low. The CVs of the measured indices ranged from 2.90% to 66.28%. Among them, pH showed the smallest variation, whereas organic matter, total nitrogen, AP, and available potassium exhibited relatively large variations, indicating that these indices were more sensitive to external disturbances (Table S1). Overall, the soils in the study area were characterized by nitrogen deficiency, potassium deficiency, and low organic matter content (Figure 6A). Spatially, soil fertility was dominated by low-fertility classes, with only a few localized patches of moderate fertility (Figure 6B), indicating that the overall soil fertility was low and its spatial variability was limited. Considering the distribution of mining areas and villages, the local differences in soil fertility showed a certain correspondence with the extent of human activities, suggesting that coal mining disturbance, surface stripping, and solid waste accumulation had adverse effects on soil quality.

To further explore the influence of soil properties on key risk elements, the relationships between soil physicochemical indices and Cd and Hg were analyzed (Figure S1). The results showed that the soil fertility indices had relatively good internal consistency, whereas Cd and Hg generally exhibited weak correlations with most physicochemical indices. Notably, organic matter was significantly negatively correlated with Cd (r = −0.26, *p* < 0.05), indicating that soil organic matter may play a certain regulatory role in the environmental behavior of Cd, and that higher organic matter levels may, to some extent, inhibit Cd migration and accumulation. In contrast, Hg showed no significant correlation with organic matter, suggesting that its enrichment was not primarily controlled by soil properties. Combined with the aforementioned source apportionment and spatial distribution results, Cd appeared to be regulated to some extent by soil properties, whereas Hg was more likely dominated by exogenous inputs, such as coal mining, coal gangue accumulation, coal combustion emissions, and atmospheric deposition. Therefore, heavy-metal pollution and its associated risks in the study area generally followed a composite pattern dominated by external inputs and regulated by soil properties.

### Human health risk assessment

**Figure 7:**
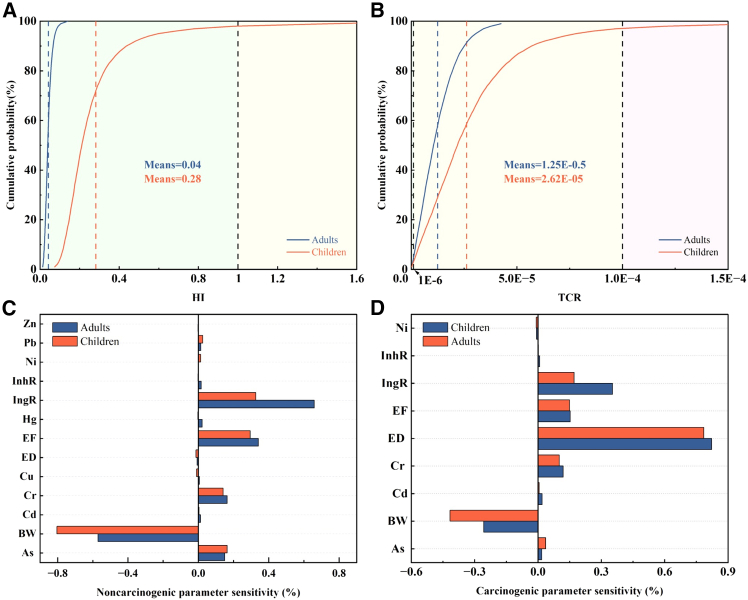
Probabilistic risk and sensitivity analysis of soil heavy metals by receptor group (A) Cumulative probability of non-carcinogenic risk (HI) for adults and children. (B) Cumulative probability of total carcinogenic risk (TCR) for adults and children. (C) Parameter sensitivity for non-carcinogenic risk. (D) Parameter sensitivity for carcinogenic risk.

To characterize the influence of parameter uncertainty on health risk assessment results, Monte Carlo simulation (MCS; 10,000 iterations) was applied to conduct a probabilistic assessment of soil heavy-metal exposure risks in the study area. Non-carcinogenic and carcinogenic risks via three exposure pathways, namely oral ingestion, inhalation, and dermal contact, were calculated separately for adults and children. The results showed that overall soil heavy-metal exposure risks in the study area were within acceptable levels, although clear differences existed among elements and between population groups. For non-carcinogenic risk (Figure 7A and Table S2), the mean hazard index (HI) values for adults and children were 0.04 and 0.28, respectively, both below the safety threshold of 1, indicating no significant non-carcinogenic health risk in the study area. In terms of elemental contributions, Cr and As showed relatively higher contributions, whereas Zn contributed the least. In addition, the HI distribution for children was overall shifted to the right relative to that for adults, indicating that children were more sensitive to soil heavy-metal exposure. For carcinogenic risk (Figure 7B and Table S3), the mean total carcinogenic risk (TCR) values for adults and children were 1.25 × 10^−5^ and 2.62 × 10^−5^, respectively, both within the acceptable range recommended by the USEPA (10^−6^–10^−4^). The contribution patterns of carcinogenic risk were generally consistent between the two population groups, following the order Cr > As > Cd > Ni, indicating that Cr and As were the major carcinogenic risk sources, and that children had higher overall carcinogenic risk than adults.

Exposure pathway analysis showed that oral ingestion was the dominant pathway for both non-carcinogenic and carcinogenic risks, followed by dermal contact, whereas inhalation contributed the least, indicating that soil particle ingestion was the key process driving health risk formation in the study area. Sensitivity analysis results (Figures 7C and 7D) further showed that non-carcinogenic risk was mainly controlled by body weight (BW), ingestion rate (IngR), and EF, whereas carcinogenic risk was primarily influenced by exposure duration (ED), followed by BW, IngR, and EF. Overall, although health risks from soil heavy metals in the study area were within acceptable limits, Cr and As remained the major risk-contributing elements, and children were the more sensitive exposed population. Therefore, future risk prevention and control should prioritize the sources of key elements and the oral exposure pathway.

## Discussion

Heavy metals are difficult to dissipate naturally, readily accumulate in surface soils, and are relatively sensitive to anthropogenic disturbance; therefore, they are commonly used as important indicators for evaluating changes in soil environmental quality, and their pollution characteristics and risk patterns can provide an important basis for regional zoning control and ecological restoration.^21^^,^^22^^,^^23^^,^^24^^,^^25^^,^^26^ Previous studies have shown that the ecological risk of soils in coal mining areas is usually dominated by elements with relatively high toxicity coefficients and strong environmental activity, such as Cd, As, and Hg^27^^,^^28^^,^^29^^,^^30^ For example, Cd has been identified as the key ecological risk factor in soils surrounding coal gangue dumps in Jilin^27^; Cd showed the highest single-factor pollution index in waste dump areas of Liupanshui, Guizhou^28^; Hg and As were the dominant ecological risk elements in open-pit coal mining areas of northern Shaanxi, followed by Cd^30^; and in coal mining areas of Xinjiang, the risks associated with Hg and Cd were also significantly higher than those of other elements.^29^ These studies indicate that although different mining areas share certain common features in terms of dominant risk elements, their risk spectra and formation mechanisms are not entirely consistent but are jointly controlled by multiple factors, including source composition, geological background, development mode, and soil physicochemical properties. Therefore, discussion of soil heavy-metal pollution in coal mining areas should not remain at the level of general result comparison but should instead be carried out in a targeted manner by integrating the specific disturbance processes, surface environmental conditions, and human activity patterns of the study area.

The study area is a typical township characterized by intensive coal mining, with dense mine distribution, long-term development disturbance, extensive coal gangue accumulation, concentrated mine transportation and coal-related combustion activities, and an interwoven spatial pattern of mining areas, villages, and farmland. In addition, it is located in a semi-arid and ecologically fragile region, with relatively low vegetation cover, high surface exposure, and active wind erosion and dust resuspension. Under these conditions, soil heavy-metal inputs were not derived from a single source or short-term event but instead reflected the coexistence of persistent exogenous inputs and multiple superimposed sources. Overall, soil heavy metals were dominated by anthropogenic sources, with natural sources ranking second. Among them, the industrial-traffic mixed source contributed the most, while the coal-related emission-atmospheric deposition source and the mining disturbance-agricultural input mixed source jointly participated in local pollution formation, indicating that coal extraction, washing, stockpiling, road transportation, and associated industrial activities may promote the accumulation of heavy metals in surface soils through dust deposition, particle resuspension, weathering-leaching, and near-surface redistribution processes.^24^^,^^31^ From the perspective of ecological risk, Cd and Hg contributed significantly more than other elements, indicating that local high-risk patches were mainly associated with the continuous input and activated accumulation of highly toxic elements. This pattern not only conforms to the general rule that highly toxic elements dominate ecological risk in mining areas^27^^,^^28^^,^^29^^,^^30^ but also reflects the composite pollution characteristics under the background of dense coal mining development. Furthermore, the controlling mechanisms of different elements within the composite system differed markedly, which is another important feature distinguishing the study area from conventional single-source contaminated sites. High-Hg zones were mainly distributed in and around mining areas and showed a certain degree of diffusive enrichment in local residential activity zones, indicating that Hg was more readily controlled by coal mining, coal combustion emissions, and atmospheric deposition. In contrast, high-Cd zones were also concentrated in and around mining areas and, in addition to local agricultural inputs, were closely related to mining disturbance processes, such as coal extraction, coal gangue accumulation, transport-related dust, and weathering-leaching.^31^ Meanwhile, soil properties regulated different elements in different ways. A higher organic matter content may enhance the adsorption and fixation of Cd, thereby suppressing its migration and redistribution, whereas Hg accumulation appeared to be more strongly controlled by continuous exogenous inputs. Overall, heavy-metal pollution and its associated risks in the study area resulted from the combined effects of exogenous input intensity, surface migration processes, and soil property regulation, and thus exhibited a composite pattern dominated by external inputs and regulated by soil properties. This mechanism not only explains why Cd and Hg were the major contributors to ecological risk in the study area but also reveals the intrinsic linkage among pollution sources, spatial differentiation, and risk responses in intensively coal-mined areas.

In addition to heavy-metal pollution, the soils in the study area also exhibited pronounced degradation characteristics, indicating that long-term mining disturbance, topsoil stripping, and wind erosion have continuously affected soil structure and nutrient status. Degraded mine lands are commonly associated with nutrient deficiency, structural damage, sparse vegetation, and an increased proportion of bare land, which not only hinder vegetation recovery and soil functional reconstruction but also weaken the buffering capacity of soils against pollutants and amplify pollution exposure risks by enhancing dust resuspension and particle dispersion processes.^32^ Soil remediation in coal mining areas is not only a practical necessity for mitigating heavy-metal pollution and restoring surface ecological functions but also an important prerequisite for safeguarding residents’ health and promoting regional sustainable development.^33^ The health risk assessment results of this study showed that oral ingestion was the dominant exposure pathway and that children were overall at higher risk than adults. Although both non-carcinogenic and carcinogenic risks remained within acceptable limits, Cr and As were still the major contributors to health risk. This finding is generally consistent with the observations of Yang et al.^25^ and Kuang et al.,^34^ who also reported that ingestion posed the highest risk and that the key health risk elements were mainly concentrated in As and Cr. Notably, the dominant elements controlling ecological risk were not identical to those driving health risk: the former was mainly driven by Cd and Hg, whereas the latter was dominated by Cr and As, indicating that different risk endpoints respond differently to pollution processes and exposure pathways. Therefore, soil environmental management in coal mining areas should not rely solely on a single concentration level or a single risk type but should instead identify priority control targets from the three dimensions of key elements, key areas, and key exposure pathways. For the present study area, ecological risk control should prioritize high-Cd and high-Hg zones and the reduction of their sources, with particular emphasis on strengthening the regulation of mine dust, stockpile disturbance, transport corridors, and coal-related emissions. In contrast, health risk prevention should place greater emphasis on As and Cr and prioritize the implementation of covering, hardening, or topsoil management measures in exposure-sensitive areas, such as zones with frequent residential activities, schools, areas surrounding residential communities, and roadsides, in order to reduce opportunities for dust contact and ingestion. Meanwhile, in combination with mine reclamation and soil quality restoration, it is necessary to enhance soil stability and vegetation recovery capacity through organic matter supplementation, soil structure improvement, and the improvement of key indicators, such as total nitrogen, AP, and available potassium.^32^^,^^33^^,^^35^ In this way, the synergistic goal of “risk reduction-ecological restoration” can be achieved. If further screening of restoration indicators is needed from the perspective of soil quality evaluation, organic matter, total nitrogen, AP, and available potassium may still be regarded as priority parameters for subsequent attention.^36^

Overall, this study demonstrates that soil heavy-metal pollution in Tongchuan Town was generally controllable but showed distinct local enrichment and risk differentiation under the combined influence of intensive coal mining and township-scale human activities. Cd and Hg were the key elements driving potential ecological risk, with high-risk areas mainly concentrated around mining sites, whereas Cr and As were the major contributors to health risk, with oral ingestion as the dominant exposure pathway and children as the more sensitive population. Source apportionment further revealed a multi-source composite pattern dominated by anthropogenic inputs, especially industrial-traffic activities and coal-related processes, while soil properties partly regulated the behavior of key elements such as Cd. These findings highlight the need for integrated management strategies that combine source reduction, exposure pathway control, and soil quality restoration to achieve coordinated ecological remediation and health risk prevention in intensive coal mining areas.

### Limitations of the study

Although this study systematically revealed the pollution characteristics, source structure, and eco-health risk responses of soil heavy metals in the study area, several limitations remain. First, the analysis was based on a single campaign of surface soil sampling and therefore did not capture the dynamic variations in heavy-metal input, migration, and transformation under different seasonal and depth conditions. Second, source apportionment was mainly based on total concentration data and the PMF model. Although the sources of key elements were comprehensively inferred by combining spatial distribution and soil physicochemical properties, the lack of more direct tracers, such as speciation, valence states, or isotopes, still introduces uncertainty into the fine discrimination of certain sources. Third, the exposure parameters used in the health risk assessment were still mainly derived from USEPA-recommended values and related literature, and were not corrected according to local residents’ activity patterns or children’s behavioral characteristics. Fourth, the present study did not explicitly classify the coal-mining area into different mining disturbance or land-damage types; therefore, the specific differences in heavy-metal accumulation, source contributions, and eco-health risks among different damage types could not be further quantified. Future studies should combine multi-season, multi-depth, and multi-medium monitoring with speciation analysis, isotope tracing, localized parameter correction, and classification of different mining-damage types to further improve the interpretation of heavy-metal pollution sources, migration processes, and risk formation mechanisms in intensively coal-mined areas.

## Resource availability

### Lead contact

Further information and requests for resources should be directed to and will be fulfilled by the lead contact, Lijing Fang (flj@imufe.edu.cn).

### Materials availability

This study did not generate new unique reagents.

### Data and code availability

• Data: Data reported in this paper will be shared by the lead contact upon request.
• Code: This paper does not report original code.
• Other items: Any additional information required to reanalyze the data reported in this paper is available from the lead contact upon request.

## Acknowledgments

This study was supported by the 10.13039/501100004763Natural Science Foundation of Inner Mongolia Autonomous Region of China (grant no. 2025MS04034), the 10.13039/501100001809National Natural Science Foundation of China (grant no. 52569009), and Project of the Inner Mongolia Yellow River Basin High-Quality Development Research Center (grant no. 26HND5).

## Author contributions

Conceptualization, S.Y.; methodology, D.D. and L.F.; formal analysis, D.D., L.F., and Y.B.; investigation, D.D. and Y.B.; data curation, D.D. and L.F.; writing – original draft, D.D. and L.F.; writing – review and editing, D.D. and L.F.; funding acquisition, D.D., L.F., and H.L.; visualization, D.D. and L.F.; supervision, S.Y. and Y.H.

## Declaration of interests

The authors declare no competing interests.

## STAR★Methods

### Key resources table

**Table:** 

REAGENT or RESOURCE	SOURCE	IDENTIFIER
**Deposited data**		

Raw and analyzed data	This paper	Available from the lead contact upon request

**Software and algorithms**		

EPA PMF Model	U.S. Environmental Protection Agency	https://www.epa.gov/air-research/positive-matrix-factorization-model-environmental-data-analyses
ArcGIS 10.8	Esri	https://www.arcgis.com/index.html
Origin 2024	OriginLab	https://www.originlab.com
Oracle Crystal Ball	Oracle	https://www.oracle.com/applications/crystalball

### Experimental model and study participant details

This study does not use experimental models.

### Method details

#### Description of the study area

Tongchuan Town is located in the northeastern part of Dongsheng District, Ordos City, Inner Mongolia Autonomous Region (Figure 1). It is situated between 110°04′32″–110°08′07″ E and 39°45′15″–39°47′05″ N, covering a total area of 546 km^2^. The elevation ranges from 1269 to 1584 m. The region is characterized by a semi-arid plateau continental climate, with an average annual precipitation of 385.7 mm (primarily concentrated from July to September), an evaporation rate of 2506.3 mm, and a mean annual temperature of 6.4°C (based on 2020–2024 data). The geomorphology is dominated by a combination of hills, ravines, and low-to-medium mountains.

The vegetation is primarily desert steppe, with psammophytes (sand-loving plants) being the dominant species. Key flora includes *Salix psammophila*, *Artemisia ordosica*, *Stipa capillata*, *Caragana korshinskii*, *Elaeagnus angustifolia*, *Achnatherum splendens*, and *Amorpha fruticosa*. The vegetation is generally characterized by a short growing season, low coverage, and a sparse, shrub-like distribution. Tongchuan is a typical industrial and mining-oriented town centered on coal extraction. There are 25 coal mines within its jurisdiction, with coal reserves totaling approximately 2.5 billion tons. The coal seams are widely distributed and possess favorable mining conditions. The available arable land area is approximately 395,000 mu (approx. 26,333 ha). Major food crops include maize and tubers, while the livestock industry primarily focuses on sheep, cattle, and poultry farming.

#### Soil sample collection and pretreatment

In this study, surface soils surrounding coal mines affected by mining activities were selected as the research object. During sample site allocation, the spatial distribution characteristics of coal mining activities and the intensity of different human activities were comprehensively considered. The sampling sites covered mining areas, villages, farmland, and transportation corridors, thereby reflecting, to a certain extent, the combined influences of mining disturbance, solid waste accumulation, and surface disruption. In accordance with the quality requirements of the *Technical Specifications for Soil Environmental Monitoring* (2018)^37^ and taking into account the local geomorphological characteristics, sampling points were systematically deployed across six administrative villages in Tongchuan Town (Figure 1). A total of 88 soil samples were collected from August to October 2024, comprising 14 samples from Tianmanliang Village, 12 from Zhijita Village, 16 from Changqing Village (including Tongchuan Village), 26 from Shenshan Village, and 20 from Chaonaoliang Village. At each site, a quincunx sampling method was employed to collect five sub-samples from the surface layer (0–20 cm) within a 1 m radius. These sub-samples were thoroughly mixed, and approximately 1 kg of the composite sample was retained via the quartering method and stored in polyethylene self-sealing bags. Simultaneously, GPS coordinates and information regarding the surrounding environment were recorded.

The concentrations of heavy metals (Cu, Zn, Cr, Ni, Pb, Cd, and Fe) were determined using Inductively Coupled Plasma Mass Spectrometry (ICP-MS, Thermo Fisher iCAP-Q), with detection limits of 0.10, 0.03, 0.02, 0.28, 1.00, 0.02, and 0.02 mg kg^−1^, respectively. Hg and As were measured via Atomic Fluorescence Spectrometry (AFS-933, Jitian), with detection limits of 1.0 × 10^−4^ mg kg^−1^ and 1.0 × 10^−3^ mg kg^−1^, respectively. For quality assurance and quality control, a standard solution calibration was performed every 10 samples, and one duplicate sample was set for every 5 samples. National standard reference material (GSS-3a) was utilized for accuracy verification. The recovery rates for all elements ranged from 89.76% to 104.65%, and the relative standard deviations (RSD) were consistently below 10%. In this study, subsequent analyses focused on Cd, Hg, As, Pb, Cr, Ni, Cu, and Zn, as these elements are key soil contamination risk-screening indicators listed in the Soil Environmental Quality—Risk Control Standard for Soil Contamination of Agricultural Land (Trial),^38^ and are also commonly recognized as critical risk elements in studies of soil pollution in coal mining areas. Fe was not treated as a target element in pollution risk assessment or health risk analysis; rather, its primary role in this study was to serve as the reference element in the enrichment factor calculation.

Soil agrochemical properties were determined according to established standard methods,^39^^,^^40^ including pH, organic matter (OM), total nitrogen (TN), total phosphorus (TP), total potassium (TK), available nitrogen (AN), available phosphorus (AP), and available potassium (AK). Specifically, soil pH was measured using a pH meter at a soil-to-water ratio of 1:5. OM was determined via the potassium dichromate titrimetric method. TN was analyzed using the Kjeldahl method. TP was determined using the perchloric acid-sulfuric acid digestion method. TK was measured via hydrofluoric acid-perchloric acid digestion. For the analysis of available nutrients, AN was quantified using the alkaline hydrolysis diffusion method. AP was determined by sodium bicarbonate extraction followed by molybdenum-antimony spectrophotometry. AK was measured through neutral ammonium acetate extraction combined with flame photometry.

#### Methods

##### Enrichment factor

The Enrichment Factor is a widely utilized environmental assessment method to characterize the degree of enrichment or depletion of specific elements in environmental media.^13^ Given that Fe is relatively stable in soil parent materials and is commonly used as a reference element in enrichment factor analysis, Fe was selected as the reference element in this study.^41^ The background values were obtained from the soil heavy metal background levels of Inner Mongolia.^17^ The classification for EF-based pollution levels is provided in Table S7.^42^ The calculation formula is as follows:

(Equation 1) $$\text{EF}=\left(\frac{C_{i}}{C_{ref}}\right)/\left(\frac{B_{i}}{B_{ref}}\right)$$

Where EF represents the enrichment factor of the element; *C*_*i*_ and *C*_*ref*_ are the concentrations of heavy metal *i* and the reference element in the sample, respectively; *B*_*i*_ and *B*_*ref*_ denote the background values of heavy metal *i* and the reference element in the earth’s crust, respectively.

#### Potential ecological risk index

The Potential Ecological Risk Index (RI), originally proposed by the Swedish researcher Hakanson (1980),^43^ is a quantitative method used to evaluate ecological risk by integrating the toxicity response coefficients of multiple heavy metals and their respective contamination levels. The calculation formulas are as follows:

(Equation 2) $$E_{j}^{i}=T_{j}\times \frac{C_{j}^{i}}{C_{j}^{0}}$$

(Equation 3) $$\text{RI}=\sum_{j=1}^{n}E_{j}^{i}$$

Where *RI* is the integrated potential ecological risk index; $E_{j}^{i}$ represents the individual potential ecological risk index for heavy metal *j*; *T*_*j*_ denotes the toxicity response coefficient for heavy metal *j*; $C_{j}^{i}$ is the measured concentration of heavy metal *j*; $C_{j}^{0}$ is the reference value for heavy metal *j*; and *n* is the number of heavy metals included in the assessment. The classification standards for $E_{j}^{i}$ and *RI* are detailed in Table S1. In this study, the soil element background values of Inner Mongolia were utilized as the reference values. Based on the Hakanson method, the toxicity response coefficients for Cd, Cr, Hg, As, Pb, Zn, Cu, and Ni are assigned values of 30, 2, 40, 10, 5, 1, 5, and 5, respectively.

In this study, the Inner Mongolia soil background values were used as the reference values for EF and RI calculations, as they can reflect regional-scale geochemical characteristics and provide relatively good comparability. However, these background values may not fully capture local geological heterogeneity within the study area, thereby introducing some uncertainty into the assessment of enrichment levels. If the local background values are lower (or higher) than the regional average, the evaluation results may be overestimated (or underestimated), respectively. It should be noted that this uncertainty mainly affects the absolute values of the assessment results, while having relatively limited influence on the relative variation trends among different elements and their spatial distribution patterns. Therefore, although the choice of background values may affect the absolute determination of pollution levels, it is unlikely to substantially alter the overall understanding of the relative enrichment characteristics, spatial differentiation patterns, and major driving sources of heavy metals in this study.

#### Soil quality evaluation

Based on the *Nutrient Classification Standards of the Second National Soil Survey*^44^ and the specific conditions of the study area, an evaluation index system was constructed comprising eight indicators: soil pH, organic matter (OM), total nitrogen (TN), total phosphorus (TP), total potassium (TK), available nitrogen (AN), available phosphorus (AP), and available potassium (AK). The soil fertility quality was comprehensively evaluated using the Modified Nemerow Integrated Index method.^45^^,^^46^ The classification standards for individual soil nutrients, individual nutrient quality, and integrated soil fertility^47^ are detailed in Tables S5 and S6. For alkaline soils (pH > 7), the pH values were first converted using the formula pH = 14 - pH before calculation with the modified Nemerow index.^48^ The calculation procedures are as follows:

(Equation 4) $$P_{i}=C_{i}/X_{1}\left(C_{i}\leq X_{1}\right)$$

(Equation 5) $$P_{i}=k+\left(C_{i}-X_{k}\right)/\left(X_{k+1}-X_{k}\right)\;\left(X_{k}\leq {C_{i}<X}_{k+1};k=1,2,3,4\right)$$

(Equation 6) $$P_{i}=5\;\left(C_{i}\geq X_{5}\right)$$

(Equation 7) $$P={\left[\left(P_{iavg}^{2}+P_{imin}^{2}\right)/2\right]}^{1/2}\times \left(m-1\right)/m$$

Where *P* represents the comprehensive soil fertility quality index; *P*_*iavg*_ is the average value of the individual quality indices; *P*_*imin*_ is the minimum value among the individual quality indices; and *m* denotes the number of evaluation indicators. *P*_*i*_ represents the individual quality index for indicator; *C*_*i*_ is the measured value of the indicator; and *X*_1_∼*X*_5_ are the critical standard values for the corresponding indicators.

#### Positive matrix factorization (PMF) model

Positive Matrix Factorization (PMF) is an effective factor analysis model widely utilized for source apportionment of pollutants in atmospheric particulates, water environments, and sediments. In this study, EPA PMF version 5.0 (2014) was employed. The model decomposes the sample concentration data matrix *x*_*ij*_ into two factor matrices-source contribution *g*_*ik*_ and source profile *f*_*kj*_ - plus a residual matrix *e*_*ij*_. The calculation formula is as follows:

(Equation 8) $$X_{ij}=\sum_{k=1}^{p}g_{ik}f_{kj}+e_{ij}$$

Where *x*_*ij*_ represents the concentration of element *j* in sample *i*; *g*_*ik*_ is the contribution of source *k* to sample *i*; *f*_*kj*_ is the concentration of element *j* in source *k*; and *e*_*ij*_ is the residual matrix, representing the portion of the concentration matrix *x*_*ij*_ that remains unexplained by the *PMF* model. *PMF* performs constrained iterative calculations based on the weighted least squares method to minimize the objective function *Q*, defined as:

(Equation 9) $$Q={\sum_{i=1}^{n}\sum_{j=1}^{m}\left(\frac{e_{ij}}{u_{ij}}\right)}^{2}$$

Where *μ*_*ij*_ is the uncertainty of the concentration of element *j* in sample *i*. If the element concentration exceeds the Method Detection Limit (*MDL*), the uncertainty (*Unc*) is calculated as follows:

(Equation 10) $$Unc=\sqrt{{\left(\delta \times c\right)}^{2}+{\left(0.5\times MDL\right)}^{2}}\;\left(c>MDL\right)$$

Where *δ* is the relative standard deviation, *C* is the element concentration (mg·kg^−1^), and *MDL* is the method detection limit for heavy metals (mg·kg^−1^). If the concentration is less than or equal to the *MDL*, the uncertainty is calculated as:

(Equation 11) $$Unc=\frac{5}{6}\times MDL\;\left(c\leq MDL\right)$$

On the basis of PCA-MLA analysis, positive matrix factorization (PMF) was applied to quantitatively apportion the source contributions of soil heavy metals. A signal-to-noise ratio (S/N) > 2 was used as the criterion for variable selection to ensure the suitability of the dataset for PMF modeling. To determine the optimal number of factors, models with 3, 4, and 5 factors were tested, and each factor solution was run 20 times with different initial values to avoid convergence to local minima. The robust Q value (Q(Robust)) was used as the primary evaluation criterion, together with residual distribution and the environmental interpretability of factor profiles. As shown in Table S4, the 4-factor model yielded stable Q(Robust) values ranging from 1504.1 to 1506.4, which were substantially lower than those of the 3-factor model (approximately 12,374, with two runs exceeding 15,850) and the 5-factor model (approximately 4,582). Standardized residual analysis showed that about 96% of the residuals had absolute values below 3, with a generally symmetric distribution and no obvious systematic bias. The results from the 20 runs were highly consistent, and the source profiles of the four factors showed clear environmental significance. Therefore, the 4-factor solution was ultimately selected as the most robust and physically interpretable result for subsequent source contribution analysis.

#### Human health risk assessment model

Heavy metal pollutants in soil can be dispersed across various sites through surface runoff and anthropogenic activities (e.g., trampling and excavation). These contaminants enter the human body via three primary exposure pathways: incidental ingestion, inhalation, and dermal contact, thereby posing potential health hazards.^49^ In this study, the non-carcinogenic and carcinogenic health risks associated with soil heavy metal accumulation were evaluated using the health risk assessment model recommended by the United States Environmental Protection Agency (USEPA). The calculation formulas are as follows:

(Equation 12) $$ADD_{ing}=\frac{C\times {IR}_{ing}\times CF\times EF\times ED}{BW\times AT}$$

(Equation 13) $$\text{AD}D_{inh}=\frac{C\times IR_{inh}\times EF\times ED}{PEF\times BW\times AT}$$

(Equation 14) $$\text{AD}D_{der}=\frac{C\times SA\times CF\times AF\times ABS\times EF\times ED}{BW\times AT}$$

(Equation 15) $${HQ}_{ij}=\frac{{ADD}_{ij}}{R{fD}_{ij}}$$

(Equation 16) $$\text{CR}=ADD_{ij}\times SF_{ij}$$

(Equation 17) TCR=∑CR

Where ADD_ing_, ADD_inh_, and ADD_der_ represent the average daily dose through incidental ingestion, inhalation, and dermal contact, respectively.^50^^,^^51^^,^^52^ The specific values for these parameters are provided in Table S8. *HQ*_*ij*_ is the hazard quotient (non-carcinogenic risk) for heavy metal *i* via exposure pathway *j*; HI is the hazard index, representing the total non-carcinogenic risk across the three pathways; *CR*_*ij*_ denotes the carcinogenic risk for heavy metal *i* via pathway *j*; and TCR is the total carcinogenic risk. The risk characterization criteria are defined as follows: an HI < 1 indicates that the non-carcinogenic risk is within an acceptable range, whereas an HI > 1 suggests the presence of non-carcinogenic health effects. Regarding carcinogenic risk, a TCR <10^−6^ implies no significant risk; a TCR between 10^−6^ and 10^−4^ indicates an acceptable or tolerable risk; and a TCR >10^−4^ indicates a significant carcinogenic risk. The values for RfD and SF are listed in Table S9.

#### Monte Carlo simulation

Monte Carlo simulation (MCS) is a probability distribution-based uncertainty analysis method that characterizes the variation range and probability distribution of exposure risk outcomes through random sampling of key parameters, thereby providing a more comprehensive reflection of the uncertainty associated with health risks induced by heavy metal exposure than conventional deterministic assessment.^53^ In addition, when combined with sensitivity analysis, MCS can further identify the key parameters that exert the greatest influence on risk outcomes, thus providing a basis for subsequent risk control and priority management.^54^^,^^55^^,^^56^

In this study, MCS was applied to the health risk assessment of heavy metals to propagate the uncertainty of input parameters. The uncertain inputs included concentration (Cj), exposure frequency (EF), exposure duration (ED), and body weight (BW). The types and ranges of parameter distributions were defined according to USEPA-recommended values and relevant literature (Tables S8 and S9). Simulations were performed in Oracle Crystal Ball v11.1.2.4 (an Excel add-in) with 10,000 Monte Carlo iterations.

### Quantification and statistical analysis

All statistical analyses were performed in ArcGIS 10.8 and Origin 2024. The specific tests used are clearly listed in the figures and tables.

### Additional resources

No additional resources are available.

## References

[1] Deng W., An Y., Huang Y., Han B., Li D., Wang Y. (2025). Heavy metal pollution assessment and health risk assessment of urban land based on soil-body system: A case study of Beijing Municipal Administrative Center. Environ. Chem..

[2] Tang H., Deng Q., Yuan Y., Zhang S., Luo Y., Chen Y., Jiang L., Huang Y. (2024). The spatial distribution and source of heavy metals in soil-plant-atmosphere system in a large coal mining area. Ore and Energy Resource Geology.

[3] Zhang Z., Lu R., Wu S., Jia Z., Wang N. (2022). Heavy metal pollution and health risk assessment of mine soil in Yangtze River economic belt. Environmental Science.

[4] Zhang Y., Cao Y., Feng N., Liu Y., Zhang Y., Wang Q., Liu J. (2024). Risk assessment of heavy metals in the soil of an abandoned coal mine area. J. China Coal Soc..

[5] Wei Z., Jiang X., Huang A., Yang Z., Zhao J., He F., Tian Y., Yan L., Xia X., Zhang Q., Jiang A. (2025). Potential ecological risk assessment of soil heavy metal pollution in typical coal mine area of Qujing, Yunnan Province. Geoscience.

[6] Paatero P., Tapper U. (1994). Positive matrix factorization: A non-negative factor model with optimal utilization of error estimates of data values. Environmetrics.

[7] Jiang H.H., Cai L.M., Wen H.H., Hu G.C., Chen L.G., Luo J. (2020). An integrated approach to quantifying ecological and human health risks from different sources of soil heavy metals. Sci. Total Environ..

[8] Jianfei C., Chunfang L., Lixia Z., Quanyuan W., Jianshu L. (2020). Source apportionment of potentially toxic elements in soils using APCS/MLR, PMF and geostatistics in a typical industrial and mining city in Eastern China. PLoS One.

[9] He Y., Han X., Ge J., Wang L. (2022). Multivariate statistical analysis of potentially toxic elements in soils under different land uses: Spatial relationship, ecological risk assessment, and source identification. Environ. Geochem. Health.

[10] Raj D., Kumar A., Maiti S.K., Tripti, Maiti S.K. (2022). Health risk assessment of children exposed to the soil containing potentially toxic elements: A case study from coal mining areas. Metals.

[11] Jiang C., Peng X., Dang Z., Li J., Dong W., Yang X., Zhang Y., Bai X., Yang Q. (2023). Temporal process and leaching characteristics for runoff pollutants in typical solid waste improved bioretention filters. Water Air Soil Pollut..

[12] Ma J., Shen Z., Zhang P., Liu P., Liu J., Sun J., Wang L. (2023). Pollution characteristics and source apportionment of heavy metals in farmland soils around the gangue heap of coal mine based on APCS-MLR and PMF receptor model. Environ. Sci..

[13] Chakraborty P., Singh S., Hazra B., Majumdar A.S., Kumari J. (2024). Spatial distribution, source apportionment, and health risks assessment of trace elements in pre- and post-monsoon soils in the coal-mining region of North Karanpura basin, India. Sci. Total Environ..

[14] Yang J., Zhou X., Wang X., Gu X., Chen Y., Gao L., Chen X. (2021). Environmental quality evaluation and quantitative tracing of heavy metals in coal mine soils in grassland based on PCA/APCS. Acta Agrestia Sinica.

[15] Zhang X., Zhang S., Lu J., Xu J., Li X., Zhang X. (2024). Risks of heavy metal contamination in soils of coal mining community and traceability based on PMF-HHR model. China Environ. Sci..

[16] Niu H., Hua Y., Ma L., Zhang S., Guan R., Sheng M., Duan W. (2025). Factors influencing heavy metal pollution in open-pit coal mine dumps. J. East China Normal Univ. (Nat. Sci.).

[17] China National Environmental Monitoring Centre (1990).

[18] Chen Y., Qu X., Zhang B., Li X., Zhu G., Cha T. (2022). Ecological and health risk assessment of heavy metal pollution in farmland soils of Xianghe County. Environ. Sci..

[19] Zhang S., Meng F., You Y., Wang S. (2023). APCS-MLR combined with PMF model for sediment heavy metal source analysis and risk assessment in the upper Tarim River Basin. Environ. Chem..

[20] Yu L., Ma H., Wang C. (2024). Characteristics, ecological risk assessment, and source apportionment of soil heavy metals in the Yellow River floodplain of Yinchuan City. Environ. Sci..

[21] Liu F., Wang X., Chi Q. (2021). Spatial variation and health risk assessment of thallium in floodplain soil in “Three Rivers” regions of southwest China. China Environ. Sci..

[22] Ma J., Tian W., Wang K., Bao X., Wang J., Cui D., Xiang P. (2021). Bioaccessibility and toxic effects of heavy metals in field soils from an electronic disassembly plant. China Environ. Sci..

[23] Xiang M., Li Y., Yang J., Lei K., Li Y., Li F., Zheng D., Fang X., Cao Y. (2021). Heavy metal contamination risk assessment and correlation analysis of heavy metal contents in soil and crops. Environ. Pollut..

[24] Cai Z., Lei S., Zhao Y., Gong C., Wang W., Du C. (2022). Spatial distribution and migration characteristics of heavy metals in grassland open-pit coal mine dump soil interface. Int. J. Environ. Res. Public Health.

[25] Yang X., Cheng B., Gao Y., Zhang H., Liu L. (2022). Heavy metal contamination assessment and probabilistic health risks in soil and maize near coal mines. Front. Public Health.

[26] Lai J., Yang D., Liu L., Ma Y., Wang Y. (2024). Characteristics and source identification of heavy metal pollution in shallow topsoil in Yinchuan City, northwest China. China Environ. Sci..

[27] Jiang X., Lu W.X., Zhao H.Q., Yang Q.C., Yang Z.P. (2014). Potential ecological risk assessment and prediction of soil heavy-metal pollution around coal gangue dump. Nat. Hazards Earth Syst. Sci..

[28] Ge H., Feng Y., Li Y., Yang W., Gong N. (2016). Heavy metal pollution diagnosis and ecological risk assessment of the surrounding soils of coal waste pile at Naluo Coal Mine, Liupanshui, Guizhou. Int. J. Min. Reclam. Environ..

[29] Fan T., Pan J., Wang X., Wang S., Lu A. (2022). Ecological risk assessment and source apportionment of heavy metals in the soil of an opencast mine in Xinjiang. Int. J. Environ. Res. Public Health.

[30] Wang N., Liu Z., Sun Y., Lu N., Luo Y. (2024). Analysis of soil fertility and toxic metal characteristics in open-pit mining areas in northern Shaanxi. Sci. Rep..

[31] Ma J., Shen Z.j., Wang S.l., Deng L., Sun J., Liu P., She Z.l. (2023). Source apportionment of heavy metals in soils around a coal gangue heap with the APCS-MLR and PMF receptor models in Chongqing, southwest China. J. Mt. Sci..

[32] Zhu S.C., Zheng H.X., Liu W.S., Liu C., Guo M.N., Huot H., Morel J.L., Qiu R.L., Chao Y., Tang Y.T. (2021). Plant-soil feedbacks for the restoration of degraded mine lands: A review. Front. Microbiol..

[33] Hou L., Hao Z., Chen B., Zhang C. (2025). Research and application prospects of bioremediation technologies for heavy metal-contaminated soil in coal mining areas in China. China Coal.

[34] Kuang Y., Chen X., Zhu C. (2024). Characteristics of soil heavy metal pollution and health risks in Chenzhou City. Processes.

[35] Wang L., Liu Q., Bai R. (2025). Soil heavy metal pollution and health risk assessment based on Monte Carlo simulation: Case study of Xicheng lead-zinc mining area. Sustainability.

[36] Jin H., Shi D., Chen Z., Liu Y., Lou Y., Yang X. (2018). Evaluation indicators of cultivated layer soil quality for red soil slope farmland based on cluster and PCA analysis. Trans. Chin. Soc. Agric. Eng..

[37] Ministry of Ecology and Environment of the People’s Republic of China (2018).

[38] Ministry of Ecology and Environment of the People’s Republic of China (2018).

[39] Lu R. (2000).

[40] Bao S. (2005).

[41] Li J., Gao Z., Ma L., Tuo X., Zhou F., Li X., Ma X., Z F. (2025). Accumulation characteristics, pollution risk, and source analysis of soil heavy metals around a coal mining area in the Baiyin section of the Upper Yellow River. Environmental Science.

[42] Sutherland R.A. (2000). Bed sediment-associated trace metals in an urban stream, Oahu, Hawaii. Environ. Geol..

[43] Hakanson L. (1980). An ecological risk index for aquatic pollution control. A sedimentological approach. Water Res..

[44] National Soil Census Office (1992).

[45] Guo C., Sun Y., Li H., Fang H., Yu Y., Liu Y. (2020). Soil nutrients assessment of ecological restoration zone of West Open Pit of Fushun Mine. J. Shenyang Agric. Univ..

[46] Cui X., Zhou Y., Liu X., Bai Z. (2021). Comprehensive evaluation of rock and soil quality of different geological stratum groups in Pingshuo opencast coal mine reclamation area. Hydrogeol. Eng. Geol..

[47] Zhang Z., Jiao J., Chen T., Chen Y., Lin H., Xu Q., Cheng Y., Zhao W. (2022). Soil nutrient evaluation of alluvial fan in the middle and lower reaches of Lhasa River Basin. J. Plant Nutr. Fertil..

[48] Ma L. (2000). Statistical data standardization: Dimensionless methods—learning and application of modern statistical analysis methods (III). Beijing Statistics.

[49] Liu Y., He Z., Niu X., Zhang D., Pan B. (2022). Health risk assessment of soil heavy metals in a small watershed of a mining area in Yunnan. Environ. Sci..

[50] Li W., Yang B., Xiong J., Zhou H., Tang H., Sun J., Lv X. (2024). Risk assessment of heavy metals in Ganqu wetland based on Monte Carlo simulation. Environ. Sci. Technol..

[51] Yang B., Xiong J., Li W., Xie P., Yang J., Huang R., Lv X. (2024). Health risk assessment of soil heavy metals in Lhasa urban area based on Monte Carlo simulation. Environ. Chem..

[52] Luo S., Yang G., Chen L., Yuan S., Yu Z. (2025). Health risk assessment of heavy metals in industrial legacy soil based on Monte Carlo simulation. Nonferrous Met..

[53] Qu L., Huang H., Xia F., Liu Y., Dahlgren R.A., Zhang M., Mei K. (2018). Risk analysis of heavy metal concentration in surface waters across the rural-urban interface of the Wen-Rui Tang River, China. Environ. Pollut..

[54] Yang S., Zhao J., Chang S.X., Collins C., Xu J., Liu X. (2019). Status assessment and probabilistic health risk modeling of metals accumulation in agriculture soils across China: A synthesis. Environ. Int..

[55] Zhou B., Zeng X., Wang Q., Liu Y., Liu X., Wu Y., Gong Z., Fang M. (2024). Exposure and health risk assessment of heavy metal in crayfish from the middle and lower reaches of the Yangtze River. Biol. Trace Elem. Res..

[56] Zhang Y., Jiang B., Gao Z., Wang M., Feng J., Xia L., Liu J. (2024). Health risk assessment of soil heavy metals in a typical mining town in north China based on Monte Carlo simulation coupled with positive matrix factorization model. Environ. Res..

