# Supplementary material for: Soil heavy metals in a typical coal mining cluster of Northern China: Pollution, source apportionment, and eco-health risks
Source: iScience. 2026 Jul 16;29(8):116835. doi: 10.1016/j.isci.2026.116835 (PMC13401030; doi:10.1016/j.isci.2026.116835)
Supplement: Document S1. Figure S1, and Tables S1–S9 [file mmc1.pdf]

iScience, Volume *29*

Supplemental information

Soil heavy metals in a typical coal mining cluster of Northern China: Pollution, source apportionment, and eco-health risks

Dandan Du, Lijing Fang, Shuyu Yu, Yanying Bai, Yusheng Hao, and Hao Li

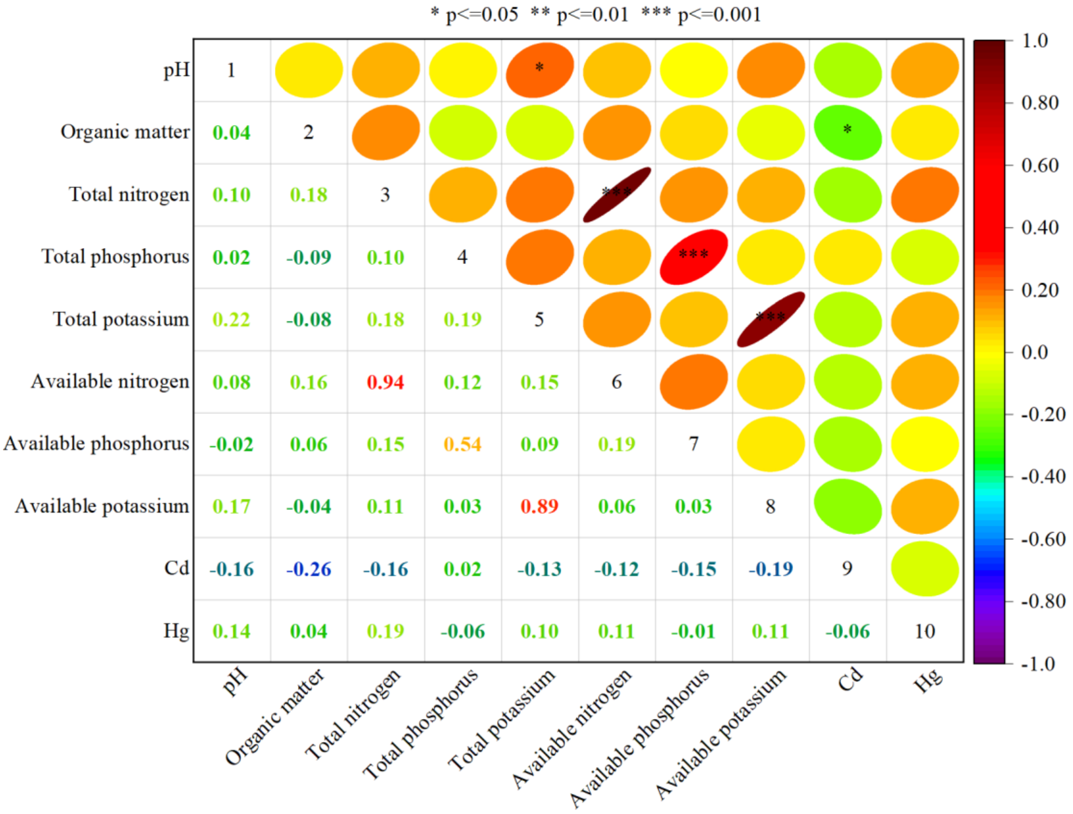

Figure S1. Correlation matrix of soil physicochemical properties and heavy metals (Cd and Hg)

Table S1. Statistics of soil nutrient content and evaluation results of individual soil fertility quality.

parameter	Statistics of soil nutrient content				Distribution of single-factor quality index grades (%)					
	Range	Mean	SD	CV	Extremely deficient I	Deficient II	Moderate III	Relatively abundant IV	Abundant V	Extremely abundant VI
pH	7.44~8.86	8.47	0.25	2.90	0	0	55.68	39.77	3.41	1.14
Organic matter	3.19~20.14	7.29	3.19	43.78	46.59	36.36	15.91	1.14	0	0
Total nitrogen	0.21~1.28	0.61	0.21	34.85	31.82	44.32	21.59	2.27	0	0
Total phosphorus	0.25~0.97	0.55	0.11	19.61	0	7.96	69.32	20.45	2.27	0
Total potassium	4.68~25.89	18.39	3.41	18.56	1.14	1.14	13.63	50.00	32.95	1.14
Available nitrogen	11.29~40.32	22.12	6.22	28.14	6.82	82.95	10.23	0.00	0	0
Available phosphorus	0.24~17.82	5.46	3.09	56.60	22.73	21.59	48.86	6.82	0	0
Available potassium	0.82~113.03	40.11	26.59	66.28	45.46	27.27	21.59	5.68	0	0

Note: pH and the coefficient of variation (CV) are dimensionless. The units for organic matter, total nitrogen, total phosphorus, and total potassium are $g \cdot kg^{-1}$, whereas those for available nitrogen, available phosphorus, and available potassium are $mg \cdot kg^{-1}$.

Table S2. Soil heavy metal non-carcinogenic risk.

Heavy Metal	Children				Adult			
	HQ_{ing}	HQ_{inh}	HQ_{der}	HI	HQ_{ing}	HQ_{inh}	HQ_{der}	HI
Cu	1.55×10^{-3}	1.20×10^{-7}	2.30×10^{-5}	1.57×10^{-3}	1.95×10^{-4}	7.93×10^{-8}	8.05×10^{-6}	2.04×10^{-4}
Zn	6.07×10^{-4}	4.71×10^{-8}	1.35×10^{-5}	6.20×10^{-4}	8.11×10^{-5}	3.31×10^{-8}	5.02×10^{-6}	8.62×10^{-5}
Pb	1.61×10^{-2}	1.25×10^{-6}	4.79×10^{-4}	1.66×10^{-2}	2.11×10^{-3}	8.58×10^{-7}	1.74×10^{-4}	2.29×10^{-3}
Cr	1.22×10^{-1}	9.97×10^{-4}	2.18×10^{-2}	1.45×10^{-1}	1.58×10^{-2}	6.78×10^{-4}	7.83×10^{-3}	2.43×10^{-2}
Cd	5.34×10^{-4}	4.12×10^{-8}	2.37×10^{-4}	7.71×10^{-4}	7.08×10^{-5}	2.88×10^{-8}	8.74×10^{-5}	1.58×10^{-4}
Ni	5.19×10^{-3}	3.91×10^{-7}	8.57×10^{-5}	5.28×10^{-3}	6.48×10^{-4}	2.57×10^{-7}	2.97×10^{-5}	6.78×10^{-4}
As	1.09×10^{-1}	5.92×10^{-4}	4.89×10^{-4}	0.11	1.35×10^{-2}	3.88×10^{-4}	1.68×10^{-4}	1.41×10^{-2}
Hg	1.39×10^{-3}	4.66×10^{-7}	8.94×10^{-5}	1.48×10^{-3}	1.89×10^{-4}	3.26×10^{-7}	3.32×10^{-5}	2.22×10^{-4}

Table S3. Soil heavy metal carcinogenic risk.

Heavy Metal	Children				Adult			
	CR_{ing}	CR_{inh}	CR_{der}	TCR	CR_{ing}	CR_{inh}	CR_{der}	TCR
Cd	$2.22\times10^{-}$	$1.32\times10^{-}$	$6.96\times10^{-}$	2.23×10^{-7}	$1.56\times10^{-}$	$6.68\times10^{-}$	$1.27\times10^{-}$	$1.43\times10^{-}$
Ni	-	$7.16\times10^{-}$	-	$7.16\times10^{-}$	-	1.58×10^{-9}	-	$1.58\times10^{-}$
As	$5.32\times10^{-}$	4.18×10^{-9}	5.76×10^{-8}	5.38×10^{-6}	$2.15\times10^{-}$	8.93×10^{-9}	$2.08\times10^{-}$	$2.37\times10^{-}$
Cr	$2.04\times10^{-}$	1.33×10^{-7}	-	2.06×10^{-5}	$9.37\times10^{-}$	2.90×10^{-6}	-	$8.66\times10^{-}$

Table S4. Classification standards for heavy metal enrichment factor and potential ecological risks.

EF		E_j^i		RI	
Range	Pollution level	Range	Risk level	Range	Risk level
<2	No/Minor enrichment	<40	Low	<150	Low
2≤EF<5	Moderate enrichment	$40\le E_j^i<80$	Moderate	150≤RI< 300	Moderate
5≤EF<20	Significant enrichment	$80\le E_j^i<160$	Considerable	300≤RI< 600	Considerable
20≤EF<40	High enrichment	$160\le E_j^i<320$	High	600≤RI < 1200	High
EF≥40	Extremely high enrichment	≥320	Very high	RI≥1200	Very high

Table S5. Classification standard of soil nutrient index.

Classification level	pH (≤7)	Organic matter OM/g·kg^{-1}	Total nitrogen TN/g·kg^{-1}	Total phosphorus TP/g·kg^{-1}	Total potassium TK/g·kg^{-1}	Available nitrogen AN/mg·kg^{-1}	Available phosphorus AP/mg·kg^{-1}	Available potassium AK/mg·kg^{-1}
X_1	4.5	6	0.5	0.2	5	30	3	30
X_2	5.0	10	0.75	0.4	10	60	5	50
X_3	5.5	20	1.0	0.6	15	90	10	100
X_4	6.0	30	1.5	0.8	20	120	20	150
X_5	6.5	40	2.0	1.0	25	150	40	200

Table S6. Classification of soil fertility quality grades.

Single-factor quality classification criteria			Comprehensive fertility classification criteria		
P_i	Rank	Quality type	P	Rank	Quality type
$P_i < 1$	I	Extremely poor	$P \leq 0.9$	I	Extremely poor
$1 \leq P_i < 2$	II	Poor	$0.9 < P \leq 1.8$	II	Poor
$2 \leq P_i < 3$	III	Moderate	$1.8 < P \leq 2.7$	III	Moderate
$3 \leq P_i < 4$	IV	Relatively rich	$2.7 < P \leq 3.5$	IV	Fertile
$4 \leq P_i < 5$	V	Rich	$P > 3.5$	V	Highly fertile
$P_i \geq 5$	VI	Extremely rich	—	—	—

Table S7. Q(Robust) values for 3, 4, and 5 factors from 20 base runs each.

Run	Q(Robust) for 3 factors	Q(Robust) for 4 factors	Q(Robust) for 5 factors
1	12373.8	1505.5	4582.46
2	12379.9	1506.21	4581.72
3	15853.9	1505.75	4582.74
4	12382.4	1505.54	4581.75
5	12374.6	1505.28	4582.99
6	12382.6	1505.16	4582.52
7	12376.3	1505.64	4581.61
8	12378.5	1505.34	4582.18
9	12377.1	1505.72	4582.39
10	12379.5	1505.35	4581.95
11	12374.6	1505.69	4583.49
12	12382.5	1505.49	4581.89
13	12377.3	1506.42	4581.7
14	12377.4	1505.47	4582.1
15	12379.7	1506.11	4582.82
16	12373.7	1506.13	4581.56
17	12384.6	1505.94	4582.61
18	12378.1	1505.69	4583.3
19	12378.2	1505.27	4583.47
20	15855.1	1504.1	4578.6

Table S8. Parameter values for health risk assessment model.

parameter	Meaning	Values	
		Children	Adults
IngR	Ingestion rate（mg·d^{-1})	Tri (66,	Tri (4, 30,
CF	Conversion factor（kg·mg^{-1})	10^{-6}	10^{-6}
ED	Exposure duration（a)	Uni (0,18)	Uni (0,
EF	Exposure frequency（d·a^{-1})	Tri (180,	Tri (180,
BW	Average body weight (kg)	Nor (36.68,	Nor
AT	Averaging time (non-carcinogenic)（d)	ED×365	ED×365
AT	Averaging time (carcinogenic) (d)	Point (70×	Point (70
InhR	Inhalation rate（m^{3}·d^{-1})	Nor (11.68,	Nor
PEF	Particle emission factor（m^{3}·kg^{-1})	Point (1.36	Point
SA	Exposed skin surface area（cm^{2})	Point	Point
AF	Skin adherence factor（mg·cm$^{2\ (-1)}$）	0.2	0.2
ABS	Dermal absorption factor（unitless)	0.001	0.001

Note: Tri = triangular distribution; Uni = uniform distribution; Nor = normal distribution; Point = fixed value.

Table S9. Reference dose and slope factor values for heavy metals.

parameter	Exposure pathway	Cu	Zn	Pb	Cr	Cd	Ni	As	Hg
Rfd	ingestion	0.04	0.3	3.5×10^{-3}	3×10^{-3}	0.001	0.02	3×10^{-4}	3×10^{-4}
	inhalation	0.0402	0.3	3.52×10^{-3}	2.86×10^{-5}	0.001	2.06×10^{-2}	4.29×10^{-6}	7.04×10^{-5}
	dermal contact	0.012	0.06	5.25×10^{-4}	7.5×10^{-5}	1×10^{-5}	5.4×10^{-3}	3×10^{-4}	2.1×10^{-5}
SF	ingestion				0.5	6.1		1.5	
	inhalation				42	6.3	0.84	15.1	
	dermal contact					6.1		3.66	
